# LRP1-Mediated Endocytosis May Be the Main Reason for the Difference in Cytotoxicity of Curcin and Curcin C on U2OS Osteosarcoma Cells

**DOI:** 10.3390/toxins14110771

**Published:** 2022-11-08

**Authors:** Siying Qin, Xueying Wang, Pan Han, Zhiping Lai, Yingying Ren, Rui Ma, Cheng Cheng, Ting Wang, Ying Xu

**Affiliations:** 1 Key Laboratory of Bio-Resources and Eco-Environment of Ministry of Education, College of Life Sciences, Sichuan University, Chengdu 610041, China; 2 The First Affiliated Hospital of Chengdu Medical College, School of Clinical Medicine, Chengdu Medical College, Chengdu 610500, China

**Keywords:** ribosome-inactivating proteins, Curcin, Curcin C, osteosarcoma U20S cells, endocytosis

## Abstract

Curcin and Curcin C, both of the ribosome-inactivating proteins of *Jatropha curcas*, have apparent inhibitory effects on the proliferation of osteosarcoma cell line U20S. However, the inhibitory effect of the latter is 13-fold higher than that of Curcin. The mechanism responsible for the difference has not been studied. This work aimed to understand and verify whether there are differences in entry efficiency and pathway between them using specific endocytosis inhibitors, gene silencing, and labeling techniques such as fluorescein isothiocyanate (FITC) labeling. The study found that the internalization efficiency of Curcin C was twice that of Curcin for U2OS cells. More than one entering pathway was adopted by both of them. Curcin C can enter U2OS cells through clathrin-dependent endocytosis and macropinocytosis, but clathrin-dependent endocytosis was not an option for Curcin. The low-density lipoprotein receptor-related protein 1 (LRP1) was found to mediate clathrin-dependent endocytosis of Curcin C. After LRP1 silencing, there was no significant difference in the 50% inhibitory concentration (IC_50_) and endocytosis efficiency between Curcin and Curcin C on U2OS cells. These results indicate that LRP1-mediated endocytosis is specific to Curcin C, thus leading to higher U2OS endocytosis efficiency and cytotoxicity than Curcin.

## 1. Introduction

Osteosarcoma is the most common primary bone malignant tumor in children and adolescents [1]. Since the 1970s, the treatment received by osteosarcoma patients has not changed [2,3]. In recent decades, many clinical trials of new drugs and research on alternative strategies of standard chemotherapy have not successfully improved the prognosis of osteosarcoma patients [3]. Therefore, exploring new efficient medicines has become a new research focus in osteosarcoma. Scientists have paid more attention to macromolecular drugs than traditional small-molecule drugs because of their efficacy and high specificity [4]. In recent years, biological macromolecular drugs accounted for about 30% of innovative drugs approved by the U.S. Food and Drug Administration, and the ratio is still rising [5].

RIPs are rRNA N-glycosylases (EC 3.2.2.22) that catalyze the elimination of a specific adenine (A4324 in rat ribosomes or the equivalent in other organisms) located in the sarcin–ricin loop (SRL) of animal 28S ribosomal RNA [6,7]. Most type I RIPs have antitumor activity and lower toxicity to intact normal cells, being the potential cancer drug candidates [8]. As early as the 1990s, TCS was found toxic to leukemia/lymphoma cells in vitro [9]. MAP30, a type I ribosome-inactivating protein extracted from *Momordica charantia*, can inhibit the proliferation of MDA MB 231 breast cancer cells that are insensitive to chemotherapy due to overexpression of HER2 [10]. Pachyerosin is a new type I ribosome-inactivating protein extracted from the seeds of *Rhamnus rhamnoides*. MTT test shows it can inhibit the proliferation of liver cancer cell HUH 7 [11]. α-MMC can block the cell cycle and inhibit the growth of lung cancer cells [12]. Curcin and Curcin C are type I RIPs found in *Jatropha curcas*. The latter has a powerful inhibitory effect on the growth of osteosarcoma U20S cells, even ten times that of Curcin [13].

Entry efficiency is one of the critical factors affecting the curative effect. Type I RIPs generally have low internalization efficiency and cytotoxicity due to the lack of a B chain with lectin activity. Moreover, their internalization pathways in mammalian cells are still less studied compared with ricin and other type II RIPs. Only a few mammalian internalization pathways of type I RIPs, such as saporin and trichosanthin, have been reported [14,15,16,17,18,19]. There is still no related report of Curcin and Curcin C. Therefore, this work tried to establish in vitro tracer systems of Curcin and Curcin C by three methods, including FITC labeling, linking eGFP tags and immunofluorescence tags and exploring their internalization efficiency, and internalization pathways and related receptors of Curcin and Curcin C into osteosarcoma U2OS cells.

## 2. Results

### 2.1. FITC-Curcin C Entered U2OS Cells More Efficiently Than FITC-Curcin

#### 2.1.1. Comparison of Three Methods for Labeling Curcin and Curcin C

To explore how Curcin and Curcin C enter U2OS cells, we first tried to establish an in vitro tracking system of Curcin and Curcin C by three methods: FITC labeling and eGFP through prokaryotic expression and immunofluorescence labeling.

**Figure 1:**
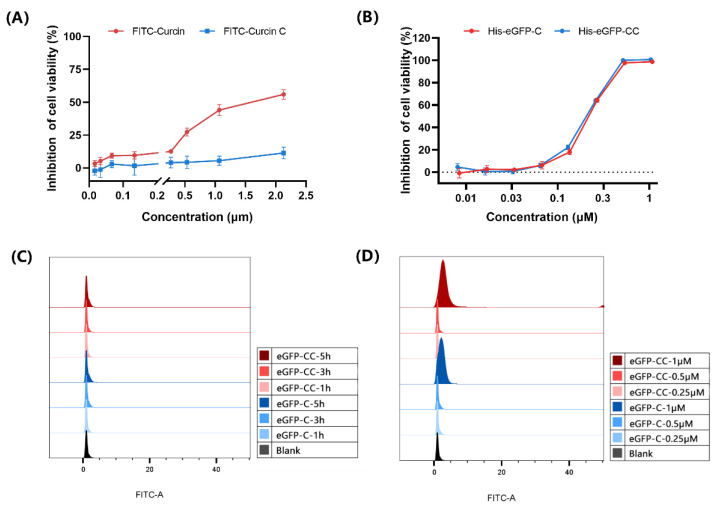
Cytotoxicity and intracellular fluorescence intensity of FITC-Curcin (FITC-C), FITC-Curcin C (FITC-CC), His-eGFP-Curcin (eGFP-C), and His-eGFP-Curcin C (eGFP-CC). (**A**) Viability of U2OS cells after 48 h FITC-C and FITC-CC treatment. (**B**) Viability of U2OS cells after 48 h eGFP-C and eGFP-CC treatment. (**C**) Flow cytometry histograms of intracellular fluorescence intensity after 1, 3, and 5 h incubation with 0.5 μM eGFP-C and eGFP-CC. (**D**) Flow cytometry histograms of intracellular fluorescence intensity after 4 h incubation with 0.25 μM, 0.5 μM, and 1 μM eGFP-C and eGFP-CC. All data are the mean ± SD of three independent experiments.

FITC labeling is a commonly used method for in vitro protein tracking, which is simple and has little effect on the structure of the target protein [20,21]. When the protein concentration was 10 mg/mL, the labeling ratio was FITC:C/CC = 70:1 and, incubated at 4 °C overnight in the dark for 12 h, the F/P value can be greater than 3, which meets the requirements of subsequent experiments. However, it is worth noting that the cytotoxicity of labeled FITC-Curcin and FITC-Curcin C were reduced. The IC_50_ values of them were 1.65 μM and greater than 2 μM (Table 1), respectively, which were much larger than those of unlabeled proteins (15.04 nM and 1.05 nM) (Figure 1A). It may be due to the presence of FITC-labeled target arginine (Arg212) in the highly conserved RNA N-glycosylase active site of Curcin and Curcin C.

Subsequently, we tried to generate His-eGFP-Curcin and His-eGFP-Curcin C using a prokaryotic expression system so that Curcin and Curcin C could be traced by eGFP tag. Although the strain expressing the recombinant vector emits specific green fluorescence of GFP, both His-eGFP-Curcin and His-eGFP-Curcin C were only obtained as inclusion bodies. Their antitumor activities after renaturation decreased by 14.38-fold and 199.35-fold, respectively (Figure 1B and Table 1). Moreover, at the experimental concentration, the fluorescence intensities of His-eGFP-Curcin and His-eGFP-Curcin C could not reach the required intensity (Figure 1C,D).

In addition, we also tried to trace His-tagged proteins (rCurcin and rCurcin C) in vitro with His-tag primary antibody and FITC-conjugated fluorescent secondary antibody. However, after many attempts, the fluorescence produced by this method was also very low and prone to false positives. Although the three in vitro tracing methods tried were not perfect, the FITC labeling method was still used in the subsequent analysis.

#### 2.1.2. FITC-Curcin C Entered U2OS Cells More Efficiently Than FITC-Curcin

**Figure 2:**
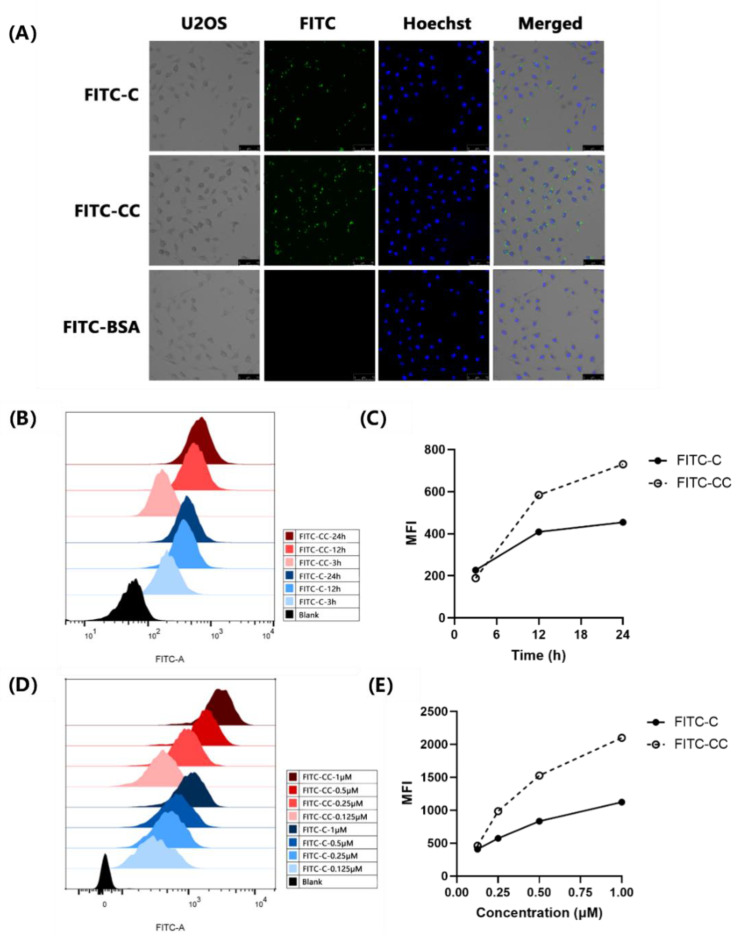
FITC-C and FITC-CC entered U2OS cells in a time- and concentration-dependent manner. (**A**) Observation of FITC-C-, FITC-CC-, and FITC-labeled bovine serum albumin (FITC-BSA) in U2OS cells under a fluorescence microscope. The nucleus was stained with DNA-specific fluorescent dye Hoechst 33258. Scale bar, 75 μm. (**B**) Flow cytometry histograms of 0.5 μM FITC-Curcin and FITC-Curcin C fluorescence intensity in U2OS cells after 3 h, 12 h, and 24 h incubation. (**C**) Mean fluorescence intensity (MFI) of 0.5 μM FITC-Curcin and FITC-Curcin C in U2OS cells after 3 h, 12 h, and 24 h incubation. (**D**) Flow cytometry histograms of FITC-Curcin and FITC-Curcin C fluorescence intensity in U2OS cells after 12 h incubation with various concentrations (0.125 μM, 0.25 μM, 0.5 μM, and 1 μM). (**E**) The MFI of FITC-Curcin and FITC-Curcin C in U2OS cells after 12 h incubation with various concentrations (0.125 μM, 0.25 μM, 0.5 μM, and 1 μM).

To visually observe whether Curcin and Curcin C can enter the cells, U2OS cells were incubated with 0.5 μM FITC-Curcin and FITC-Curcin C for 4 h, respectively. When observed under a fluorescence microscope, it is easy to detect that green fluorescence did appear inside the cells, mainly distributing in the cytoplasmic region. The results suggested Curcin and Curcin C entered the cells (Figure 2A). Meanwhile, FITC-labeled bovine serum albumin (FITC-BSA) was used as a control and was not observed entering U2OS cells.

When the concentration of FITC-Curcin and FITC-Curcin C was 0.5 μM, the amount of FITC-Curcin and FITC-Curcin C entering U2OS cells showed time-dependent laziness with the prolongation of incubation time (Figure 2B,C). When the incubation time was 12 h, the entry of FITC-Curcin C was 50% higher than that of FITC-Curcin. Then, the incubation time was fixed (12 h), and it was found that the amount of FITC-Curcin and FITC-Curcin C entering U2OS cells also showed a dose-dependent manner. When the concentration reached 1 μM, the influx of FITC-Curcin C was almost twice that of FITC-Curcin (Figure 2D,E).

### 2.2. FITC-Curcin and FITC-Curcin C Have Different Entry Pathways

The effects of six different endocytosis inhibitors on the cytotoxicity of Curcin and Curcin C were determined further to understand the reasons for the difference in entry efficiency.

**Figure 3:**
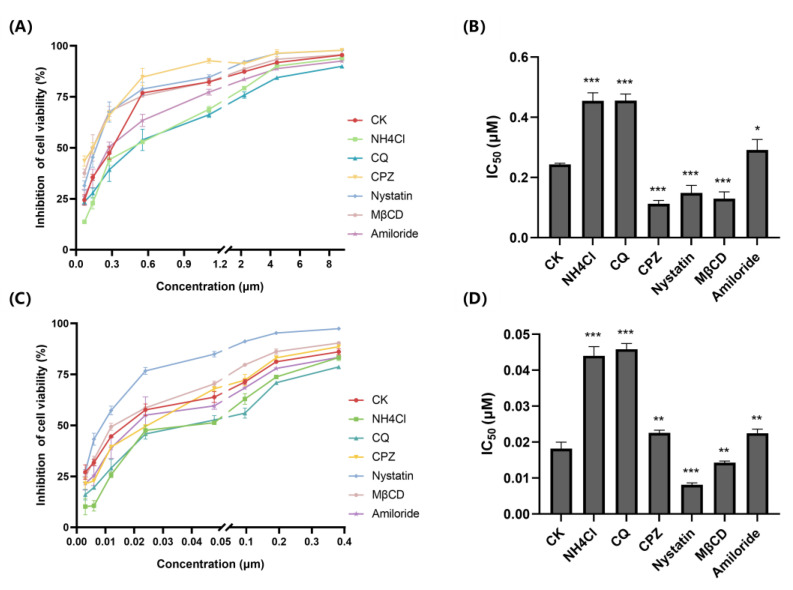
Effects of different endocytosis inhibitors on the Curcin and Curcin C cytotoxicity against U2OS cells. Control check (CK); chloroquine (CQ); chlorpromazine (CPZ); methyl-β-cyclodextrin (MβCD). (**A**) Proliferation inhibition rate of different endocytosis inhibitor-pretreated U2OS cells after 48 h Curcin treatment. (**B**) IC_50_ of Curcin after 48 h Curcin treatment on different endocytosis inhibitor-pretreated U2OS cells. (**C**) Proliferation inhibition rate of different endocytosis inhibitor-pretreated U2OS cells after 48 h Curcin C treatment. (**D**) IC_50_ of Curcin C after 48 h Curcin C treatment on different endocytosis inhibitor-pretreated U2OS cells. All data are presented as the mean ± SD of three independent experiments (***, *p* < 0.001; **, *p* < 0.01; * *p* < 0.05).

To assess whether the entry of Curcin and Curcin C into U2OS cells depends on pH-dependent endocytic pathways, we first studied the effects of two lysosomotropic agents, chloroquine (CQ) and NH_4_Cl [22]. Compared with the control, the treatment significantly reduced the proliferation inhibition rates of both Curcin and Curcin C, resulting in a significant increase in their IC_50_, suggesting that endocytosis was indeed their entry point (Figure 3).

Chlorpromazine (CPZ), a cationic amphiphilic drug, inhibits the clathrin-dependent endocytic pathway [23]. CPZ pretreatment also significantly increased the IC_50_ of Curcin C compared with the control group without inhibitor treatment. However, the increase was still lower than that in the *NH*_4_*Cl* and chloroquine (CQ) pretreatment groups (Figure 3D), suggesting the clathrin-dependent endocytosis pathway was only one of the entry pathways of Curcin C. Especially, when the concentration of Curcin C increased, the inhibition rate of cell proliferation in the CPZ pretreatment group was slightly higher than that in the control group. It might be because the clathrin-dependent endocytic pathway may be the preferred choice for Curcin C. However, when the amount of Curcin C exceeds the carrying capacity of the pathway, cells enable other pathways to transport Curcin C. Interestingly, after incubation with CPZ, the proliferation inhibition rate of Curcin at each concentration was higher than that of the control group (Figure 3A), and the IC_50_ of Curcin decreased obviously (Figure 3B), indicating that Curcin should not enter U2OS cells through a clathrin-dependent endocytic pathway. The decreased IC_50_ may be because cells’ normal growth and development are affected, and the resistance to Curcin is reduced after the clathrin-dependent endocytic pathway is inhibited.

The clathrin-independent endocytosis pathways include caveolin-dependent endocytosis, caveolin-independent endocytosis, and micropinocytosis [24]. The boundaries between the first two pathways are not very clear. Nystatin and methyl-β-cyclodextrin (MβCD) can prevent cholesterol, sphingolipids, and caveolin from invaginating on the cell membrane by removing cholesterol from the cell surface and inhibiting caveolin-dependent endocytosis [22,25]. Compared with the control, the IC_50_s of Curcin and Curcin C were significantly decreased after pretreatment of U2OS cells with Nystatin (Nystatin) and methyl-β-cyclodextrin (MβCD), indicating that the caveolin-dependent endocytosis is not the way for them to enter the cells.

Amiloride can inhibit the macropinocytosis-mediated endocytosis pathway by inhibiting Na+/H+ exchange and lowering the submembrane pH [26]. Compared with the control group, Amiloride treatment resulted in a significant increase in the IC_50_ of Curcin and Curcin C, but lower than those of the *NH*_4_*Cl* and chloroquine (CQ) pretreatment groups, which proved that macropinocytosis-mediated endocytosis is also one of the pathways by which they enter cells. However, it is worth noting that when the concentration of Curcin was lower than 0.6 μM, the proliferation inhibition rate of the Amiloride treatment group was slightly lower than that of the control group. Only when the concentration was higher than this, the inhibition rate of proliferation was lower than that of the control group, suggesting that Curcin may not enter cells through macropinocytosis at lower concentrations. Another unknown pathway might have mediated its entry.

### 2.3. LRP1 Is One of Curcin C Endocytic Receptors

#### 2.3.1. Correlation Analysis of LRP1 Abundance with Curcin and Curcin C Cytotoxicities

**Figure 4:**
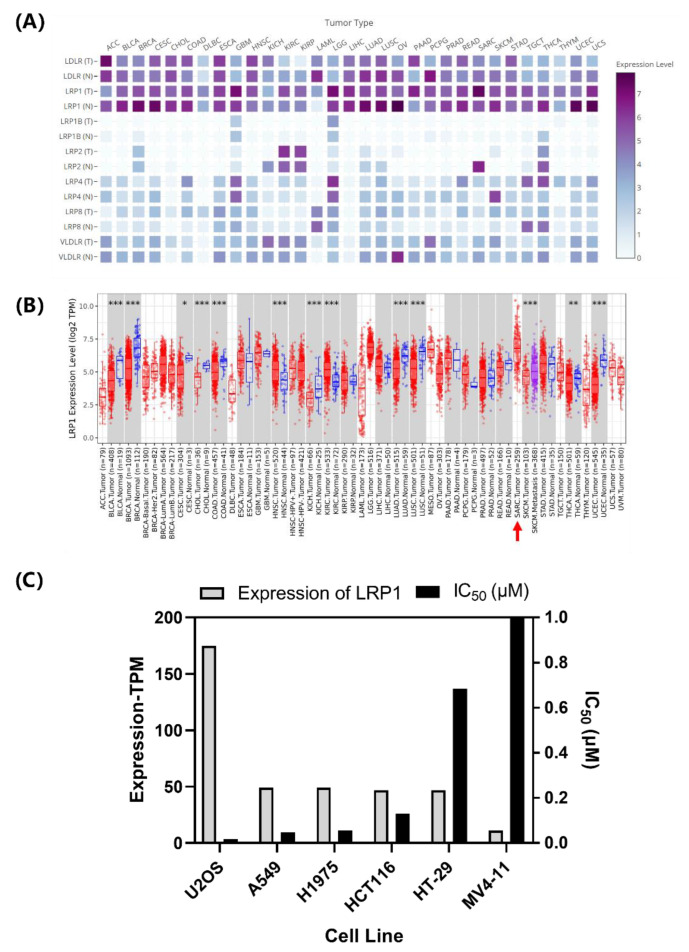
Expression of LDL family and LRP1 in tumor tissues and the relationship between LRP1 and cytotoxicity of Curcin C. (**A**) Heat map of LDL receptor family expression in 30 types of tumors and adjacent normal tissues, T—tumor tissues; N—normal tissues. (**B**) Expression of LRP1 in 35 tumor types and their adjacent normal tissues. The red arrow indicates the expression of LRP1 in sarcoma. (***, *p* < 0.001; **, *p* < 0.01; * *p* < 0.05). (**C**) Expression of LRP1 in 6 cell lines and IC_50_ of Curcin C for their corresponding cell lines. These 6 cell lines belong to 4 tumors (SARC: U2OS; LUAD: A549, H1975; COAD: HCT116, HT-29; LAML: MV4-11). IC_50_ data from Zhang et al., 2017 and Wang et al., 2022 [13,30]; the average of three independent experiments.

The above studies show that the clathrin-dependent pathway significantly differed between the Curcin and Curcin C endocytic pathways. The low-density lipoprotein receptor family is one of the most representative receptor families in the clathrin-dependent pathway [27]. Many studies have shown that the low-density lipoprotein receptor family, especially the LRP1 receptor, mediates the endocytosis of type I RIPs [14,15,16,17,18,28,29]. To understand whether LRP1 is also involved in the transport of Curcin and Curcin C, an analysis was first performed using databases TIMER2.0 and GEPIA2 to analyze the abundances of seven central LDL family members in 30 tumor cells and adjacent normal tissues (Figure 4A). The endocytic receptor LRP1 was found to have the highest expression in human sarcoma tumor cells to which U2OS cells belong (Figure 4B). Moreover, its levels in various tumor cells were found to correlate with the IC50s of Curcin C (Figure 4C). That is, the higher the level of LRP1, the lower the IC_50_ of Curcin C. However, this correlation was not observed when it concerned Curcin (data not shown). Thus, it may be speculated that LRP1 may only be involved in the endocytosis of Curcin C and is the main reason for the difference in entry efficiency between the two (IC_50_ data are derived from previous laboratory research data [13,30]).

#### 2.3.2. LRP1 Gene Silencing Partially Reduces the Cytotoxicity of Curcin C to U2OS Cells

**Figure 5:**
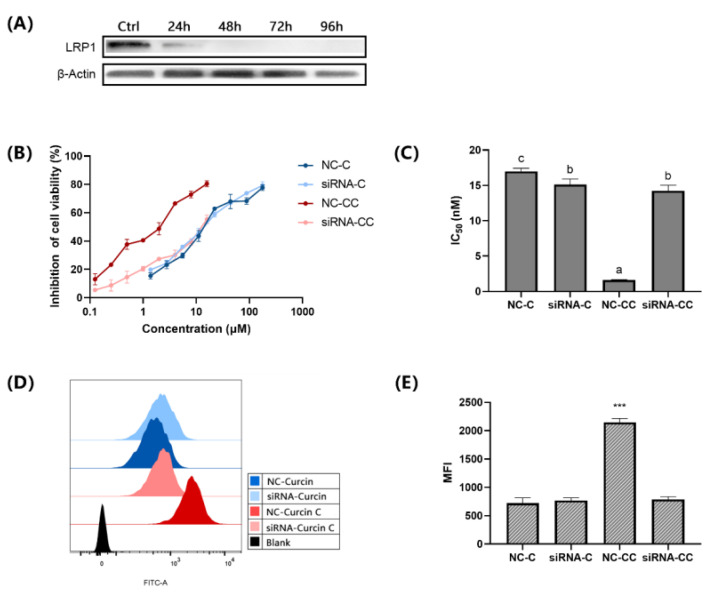
LRP1 is one of the Curcin C endocytic receptors. (**A**) Western Blot analysis of LRP1 expression levels at different time points after siRNA silencing. (**B**) Proliferation inhibition rate of siRNA-treated cells vs. untreated cells after 48 h treatment with different concentrations of Curcin and Curcin C. The “siRNA-C” and “siRNA-CC” indicate that cells were pretransfected with LRP1-siRNA for 48 h and then treated with C/CC for 48 h, and “NC-C” and “NC-CC” represent cells as the negative control, which are pre transfected with NC-siRNA for 48 h, and then treated with C/CC for 48 h. (**C**) IC_50_ of Curcin and Curcin C after 48 h of Curcin and Curcin C treatment in U2OS cells with different concentrations. (**D**) Flow cytometry was used to detect the fluorescence intensity of FITC-Curcin and FITC-Curcin C after LRP1 silencing in U2OS cells. Cells not treated with Curcin and Curcin C protein as a blank group. (**E**) The MFI of FITC-Curcin and FITC-Curcin C after LRP1 silencing vs. NC-C and NC-CC. All data are the mean ± SD of three independent experiments (***, *p* < 0.001). Different letters (a–c) indicate significant differences (*p* < 0.05; one-way ANOVA with Tukey’s honestly significant difference test).

To verify whether LRP1 was involved in the transport of Curcin C, siRNA transfection was used to silence LRP1 of U2OS cells. Western Blot detection showed that there was still a tiny amount of LRP1 24 h after transfection, but no LRP1 protein could be detected when transfected for 48 h to 96 h (Figure 5A).

The toxicity of different concentrations of Curcin and Curcin C to U2OS cells 48 h after transfection was determined. It was found that the IC_50_ of Curcin C increased significantly. After 48 h of incubation, there was an approximately 9.13-fold increase. In contrast, the IC_50_ value of Curcin decreased slightly, indicating silencing the LRP1 even enhanced the toxicity of Curcin to U2OS cells. A notable phenomenon is that after the LRP1 was silenced, the IC_50_ of Curcin and Curcin C were very close to U2OS cells, and there was no significant difference (Figure 5B,C).

#### 2.3.3. Silencing LRP1 Partially Inhibits the Entry of FITC-Curcin C into U2OS Cells

Flow cytometry was used to measure the fluorescence after U2OS cells incubated with 1 μM FITC-Curcin and FITC-Curcin C for 12 h to explore the effect of LRP1 silencing on the entry of FITC-Curcin and FITC-Curcin C into U2OS cells (Figure 5D). In the negative control group, the average fluorescence intensity of FITC-Curcin C was 2.97 times that of FITC-Curcin, indicating that the amount of FITC-Curcin C entering cells was higher than that of FITC-Curcin. When the LRP1 of U2OS cells was silenced, the mean fluorescence intensity of FITC-Curcin C decreased significantly but was not substantially different from that of FITC-Curcin (Figure 5E). It indicates that LRP1 silencing prevented FITC-Curcin C from entering U2OS cells. Considering there was no effect on the entry of FITC-Curcin into U2OS cells, the result suggests that the difference in the endocytosis receptors LRP1 may be one of the main reasons for the difference in endocytosis efficiency between Curcin and Curcin C.

## 3. Discussion

The amino acid composition and three-dimensional structure of Curcin and Curcin C are similar, but their cytotoxicity to osteosarcoma cell U2OS is 14 times different [13]. The in vitro tracer system and endocytosis inhibitors demonstrated that there might be multiple pathways involved in their transport and that the LRP1-mediated endocytic pathway might be the main reason for the difference in cytotoxicity to osteosarcoma U2OS.

Internalization efficiency primarily affects the efficacy of molecular drugs [14,15,29,31]. Due to the presence of B-chain lectins, type II RIPs possess high cell entry efficiency, leading to a dramatic increase in toxicity [32,33]. Conese et al. also found evidence that LRP1 can mediate saporin endocytosis in U937 cells, and downregulation of LRP1 can reduce the sensitivity of U937 cells to urokinase–saporin conjugates [14]. Our results also proved that though both FITC-Curcin and FITC-Curcin C could enter U2OS cells in a time- and concentration-dependent manner, the endocytosis efficiency of FITC-Curcin C was significantly higher than that of FITC-Curcin. These results suggest that it is likely that the difference in endocytosis efficiency is responsible for such significant activity between the two.

There are multiple routes for protein drugs to enter cells. For the entry of RIPs, research showed that there is also more than one pathway [34]. For example, in the endocytosis of ricin, the involved pathways include clathrin-dependent and clathrin-independent pathways. The ricin can bind to multiple receptors before it enters cells [35,36,37]. Shiga toxin can enter the cell by inducing invaginations at the cell surface [38]. Although lacking the B chain’s help, Type I RIPs such as trichosanthin can still be transported into trophoblasts and proximal tubule epithelial cells through LRP1-mediated and megalin-mediated pathways, respectively [29,31]. It is reported that the membrane insertion pathway mediated by low pH also plays a role in the intracellular transport of trichosanthin [39]. The results in this study also support that LRP1 is one of the significant pathways that mediate the entry of Curcin C into cells. Moreover, as the cytotoxicity and entry of Curcin and Curcin C were not significantly different when LRP 1 was silenced in U2OS cells, it indicates that LRP1 plays an essential role in the efficient entry of Curcin C into U2OS cells and is most likely the main reason for the difference in the activity of Curcin C and Curcin. However, the silencing of LRP1 did not reduce the entry efficiency of Curcin, and the activity of Curcin C was not completely inhibited at the same time, suggesting that there must be other endocytic pathways involved in the entry of them in addition to the LRP1-mediated pathway.

Some RIPs such as ricin enter the endocytic pathway and travel backward from the Golgi complex to the ER, which is thought to parasitize the ER-associated degradation (ERAD) pathway [32]. CQ is a weak base that accumulates inside acidic subcellular compartments, e.g., endosomes, lysosomes, and Golgi vesicles [40]. It remains trapped in a protonated state, causing an increase in pH, and thereby inhibiting the functions of these cellular compartments. We found that the CQ treatment affected the cytotoxicity of Curcin and Curcin C, implying that the intracellular transport of Curcin and Curcin C may depend on the Golgi apparatus [15]. This situation is opposite to saporin [15] but similar to ricin [32,41], which might be related to the closer evolutionary relationship between Curcin and ricin.

LRP1 not only mediates endocytosis but also plays a role in signaling. The downstream proteins include JNK pathway-related proteins, human amyloid beta (Aβ), precursor protein-binding family B member 1, Disabled-1 protein (DAB1), dense postsynaptic zone protein-954, and other proteins [42]. These proteins regulate cell proliferation and apoptosis through the mitogen-activated protein kinases (MAPK) pathway [43]. Several RIPs, including ricin A, Korean mistletoe lectin II, TCS, and Shiga toxin, induce apoptosis by activating the MAPK pathways (SAPK/JNK) [43]. Our previous study also found that Curcin C can induce apoptosis in U2OS cells by activating JNK and inhibiting the ERK signaling pathway [30]. However, further experimental evidence is needed to confirm whether Curcin C activates the JNK signaling pathway through the LRP1 receptor.

In summary, there should be multiple pathways involved in the endocytosis of Curcin and Curcin C, and LRP1 might be the main reason for the difference in activity between the two. However, we also need to provide direct evidence for the interaction between Curcin C and LRP1 through bio-layer interferometry (BLI), surface plasmon resonance (SPR) or other protein interaction technologies. At the same time, the possible endocytic receptors that mediate the entry of Curcin and Curcin C into U2OS cells via macropinocytosis are also worthy of further investigation. Moreover, the entry amount of FITC-Curcin C is only about twice that of FITC-Curcin, which is insufficient to explain the activity difference on the order of magnitude. Different entry pathways trigger various downstream events, so these mechanisms may have efficiency differences. Therefore, understanding the intracellular events triggered differently can provide more valuable clues for answering questions.

## 4. Materials and Methods

### 4.1. Materials and Cell Lines

The mature seeds of *Jatropha curcas* used in this work were collected from Panzhihua City, Sichuan Province and cultivated in our laboratory (Sichuan University, Chengdu, China). They were soaked in distilled water for 2 h and germinated in phytotron under 16/8 h day/night photoperiods at 30 °C with 1:1 mix of coconut soil and nutrient soil until the cotyledons were expanded totally, and quantitative distilled water was added daily to keep seedlings growing.

Human osteosarcoma cells U2OS were purchased from the National Collection of Authenticated Cell Cultures, and the cells were maintained in McCoy’s 5A medium (Gibco, Australia) supplemented with 10% fetal bovine serum (Cellmax Co., Ltd. (Beijing, China) and cultured in a CO_2_ incubator (Shanghai Lishen Scientific Instrument Co., Ltd. (Shanghai, China)) at 37 °C and 5% CO_2_.

### 4.2. FITC Labeling of Curcin, Curcin C, and BSA

Curcin, Curcin C, and BSA solutions were prepared by dissolving in the reaction solution (100 mM NaHCO_3_, 10 mM Na_2_CO_3_, 125 mM NaCl, and pH = 9.8) to achieve a concentration of 10 mg/mL. A total of 10 mg/mL of FITC working solution was prepared in DMSO. The volume of FITC solution was determined by the following substance ratio:FITC:BSA = 10:1 and FITC:Curcin/Curcin C = 70:1. The FITC solution was added to the protein solution slowly, while shaking to mix the solution after every 10 μL of FITC was added until all the required FITC solution was added. The resulting solution was further mixed by shaking for 12 h at 4 °C in dark. The reaction was terminated by adding 1% of the total volume of termination solution (5M NH_4_Cl) to the reaction system and then shaking for 2 h at 4 °C in dark. The solution was subsequently ultrafiltered 8 times using 10 kDa ultrafiltration tubes to remove unbound FITC from the solution. Protein concentration was determined by the Bradford protein assay kit. F/P was calculated by the following equation: F/P = A495 * Dilution factor * Path length correction/68,000 * Protein concentration (M).

### 4.3. Induced Expression of His-eGFP-Curcin and His-eGFP-Curcin C in Prokaryotes

The amino acid sequences of eGFP, Curcin, and Curcin C were obtained from the NCBI database. Codon optimization was carried out according to the genetic codon preference of *Escherichia coli*. The whole gene synthesis was carried out by Zoonbio Biotechnology. Briefly, His-eGFP-Curcin and His-eGFP-Curcin C genes were cloned into expression vectors pET30a and pCZN1 after codon optimization. The expression plasmids were transformed into the expression strains BL21 (DE3), Arctic Express, Rosetta, Shuffle T7-B, and Shuffle T7-K12. The expression was induced by different concentrations of IPTG, different temperatures, and different induction times, and the expression results were validated by SDS-PAGE. After successful induction, the expressed protein of interest was extracted using Ni column, and the supernatant was directly dialyzed into 1 × PBS (pH 7.4) at 4 °C. After dialysis, the supernatant protein was sterilized by filtering against a 0.22 μM filter head, and the resulting protein solution was aliquoted and stored at −80 °C. The inclusion bodies were dialyzed into inclusion renaturation buffer (1 × PBS (pH 7.4), 4 mM GSH, 0.4 mM GSSG, 0.4 M L-Arginine, 1 M Urea) at 4 °C for renaturation. After renaturation, the protein was dialyzed into 1 × PBS (pH 7.4). After dialysis, the protein was sterilized by filtering through the 0.22 μM filter heads. The protein was then aliquoted and stored at −80 °C.

### 4.4. Fluorescent Microscopy Observation of FITC-Curcin/Curcin C and His-eGFP-Curcin/Curcin C Entry into Cells

U2OS cells at logarithmic growth stage were seeded at 6 × 10^4^ cells/mL in a 6-well plate (2 mL per well) and incubated overnight at 37 °C with 5% CO_2_ to allow for the cells to adhere. The cells were then incubated with FITC-Curcin/ Curcin C and His-eGFP-Curcin/ Curcin C medium at the corresponding concentrations for a certain period of time, respectively. After incubation, the medium was removed, and the cells were washed 3 times with PBS. The nuclei were stained with Hoechst 33258 (Beyotime Biotech (Shanghai, China) Co., Ltd.) for 10 min at 37 °C. The cells were then washed 3 times with PBS, and then the complete medium was added. The cells were visualized using an inverted fluorescent microscope (Leica Microsystems (Wezlaer, Germany) Co., Ltd., Leica DMi 8).

### 4.5. Flow Cytometry of FITC-Curcin/Curcin C and His-eGFP-Curcin/Curcin C in Cells

U2OS cells at logarithmic growth stage were seeded at 1 × 10^5^ cells/mL in a 6-well plate (2 mL per well) and incubated overnight at 37 °C with 5% CO_2_ to allow for the cells to adhere. The cells were then incubated with FITC-Curcin/ Curcin C and His-eGFP-Curcin/ Curcin C medium at the corresponding concentrations for a certain period of time, respectively. After incubation, the medium was removed, and the cells were washed 3 times with PBS. The cells were then dissociated with 0.25% trypsin (Hyclone (Logan, UT, USA) Co., Ltd.) at 37 °C for 3 min (250 μL per well). The digestion was stopped by adding an equal amount of complete medium. The cells were dislodged by blowing, and the dislodged cell suspension was transferred into 1.5 mL EP tubes and centrifuged at 800 rpm for 3 min. The supernatant was discarded, and the cells were resuspended with 0.5 mL PBS for flow cytometry (BD (New York, NY, USA) Co., Ltd., BD LSRfortessa).

### 4.6. U2OS Cell Endocytic Inhibitors Pretreatment

U2OS cells at logarithmic growth stage were seeded at 6 × 10^4^ cells/mL in a 96-well plate (100 μL per well) and incubated overnight at 37 °C with 5% CO_2_ for cell adhesion. The cells were then pretreated for 2 h with media supplemented with 1 M MβCd, 100 mM NH_4_Cl, 50 μM CPZ, 250 mM CQ, 125 μM Amiloride, and 100X diluted Nystatin (Sigma (Shanghai, China) Co., Ltd., Topscience (Shanghai, China) Co., Ltd.). Media without any inhibitor supplemented were used as control group. The cells were subsequently treated with different concentrations of Curcin C for 48 h. Cell viability was determined by CCK-8 assay.

### 4.7. CCK8 Assay for Cell Viability Characterization

U2OS cells at logarithmic growth stage were seeded at 6 × 10^4^ cells/mL in a 96-well plate (100 μL per well) and incubated overnight at 37 °C with 5% CO_2_ for cell adhesion. Eight concentration gradients of Curcin and Curcin C protein were introduced to the cells. In addition, a blank group containing only medium and a control group without Curcin or Curcin C were set up; 5 wells of cells were used in each treatment condition. After 48 h of drug treatment, CCK8-assay was performed by culturing the cells with 10% CCK8 (Dingyou Biotech Co., LTD (Chengdu, China) at 37 °C for 1–2 h until the A450 of the control group was about 1.0. The absorbance value (A) of each group at 450 nm was measured by a microplate reader (proliferation inhibition rate = ((1 − (A of administration group—A of blank group))/(A of control group—A of blank group)) × 100%). The IC_50_ of the drug was calculated by Quest Graph™ IC_50_ Calculator.

### 4.8. LRP1 Gene Silencing in U2OS Cells by siRNA

U2OS cells at logarithmic growth stage were seeded at 6 × 10^4^ cells/mL in a 6-well plate (2 mL per well) and incubated overnight at 37 °C with 5% CO_2_ for cell adhesion. For each well, the transfection reagent was prepared by adding 100 pmol siRNA (Sangon Biotech Co., Ltd. (Shanghai, China)) into 125 μL serum-free 1640 media. Then, 3 μL Lipo8000™ transfection reagent (Beyotime Biotech Co., Ltd. (Shanghai, China)) was added. The mixture was gently mixed and incubated for 20 min at 25 °C. A total of 125 μL of the transfection reagent was added into each well, and the plate was shaken briefly to mix the reagent. After 6–8 h of culture, the medium was changed to McCoy’s 5A medium with 1 × antibiotics and 10% serum. After culture of 24 h, 48 h, 72 h, and 96 h, total protein was extracted and Western Blot was used to verify the silencing effect of LRP1. Three siRNAs were designed for desired silencing efficiency (Table 2).

### 4.9. Western Blotting for LRP1 Silencing Efficiency Characterization in U2OS Cells

After transfecting cells with siRNA in 6-well plates for 24 h, 48 h, 72 h, and 96 h, the cells were digested with 0.25% trypsin, and the digestion was stopped with an equal volume of complete medium. The cell suspension was centrifuged at 1000 rpm for 5 min at 4 °C, and the supernatant was discarded. The cells were then resuspended in 1 mL PBS. The resulting cell suspension was centrifuged again, and supernatant was discarded. For each well, the cells were lysed by adding 100 μL cell lysis buffer, 1 μL phosphatase inhibitor, and 1 μL protease inhibitor (Sigma (Shanghai, China) Co., Ltd.) and incubated at 4 °C for 10 min, followed by shaking for 15 s on ice 3 times. The cell lysates were centrifuged at 1500 RPM at 4 °C for 30 min, and the supernatant was collected and measured by Bradford protein detection kit (Sangon Biotech Co., Ltd. (Shanghai, China)). Protein concentration from each sample was normalized and separated by SDS-PAGE. The protein was transferred to PVDF membrane at 100 V for 90 min, blocked with rapid blocking solution (Beyotime Biotech (Shanghai, China) Co., Ltd.) at 25 °C for 30 min, and washed with 1X TBST for 10 min 3 times. Primary antibody was diluted 1:1000 in 5% skim milk powder and incubated overnight at 4 °C (primary antibody was LRP1, β-actin, Abways Tech Co., Ltd. (Shanghai, China)). The membrane was washed with 1× TBST for 10 min 3 times. The secondary antibody labeled with horseradish peroxidase was diluted 1:5000 in 5% skim milk powder and then incubated for 1–2 h at 25 °C. The membrane was washed with 1X TBST for 10 min × 3 times. The bands were visualized by ECL.

### 4.10. Data Analysis and Processing

All experiments were performed in biological triplicates, and the results were plotted as the mean value. GraphPad Prism 8, IBM SPSS Statistics 24, Excel, and Photoshop 2019 were used for data plotting and analysis. By one-way analysis of variance, *p* < 0.05 was defined as significant difference and marked with “*”, *p* < 0.01 was defined as highly significant difference and marked with “**”, and *p* < 0.001 was marked with “***”.

## Figures and Tables

**Table 1:** IC_50_^1^ of C ^2^, CC ^3^, C, and CC with different tags.

Protein	Curcin	Curcin C	FITC-C	FITC-CC	His-eGFP-C	His-eGFP-CC
IC_50_ (nM)	15.04	1.05	1650.24	>2000.00	216.28	209.32

^1^ IC—inhibitory concentration; ^2^ C—Curcin; ^3^ CC—Curcin C.

**Table 2:** siRNA ^1^ sequences.

Number	Gene Name	Sequence (5′-3′)
1	hLRP1-3430	CCUGCAACAAUGGCAGAUGUATT
2	hLRP1-9620	GUCCAACUACACGUUACUUAATT
3	hLRP1-4970	GCGAACAAACACACUGGCUAATT

^1^ siRNA was prepared by Sangon Biotechnology (Shanghai, China) Co., LTD.

## Data Availability

No new data were created or analyzed in this study. Data sharing is not applicable to this article.
